# Side‐to‐side characterisation of cellular content, soluble factors and in vitro potential on chondrocytes for bone marrow aspirate concentrate and adipose‐derived stromal vascular fraction

**DOI:** 10.1002/jeo2.70254

**Published:** 2025-05-12

**Authors:** Enrico Ragni, Michela Taiana, Caterina Visconte, Elizaveta Kon, Simona Landoni, Cecilia Colombo, Laura Mangiavini, Giuseppe Peretti, Laura de Girolamo

**Affiliations:** ^1^ IRCCS Ospedale Galeazzi—Sant'Ambrogio, Laboratorio di Biotecnologie Applicate all'Ortopedia Milano Italy; ^2^ IRCCS Humanitas Research Hospital Rozzano Italy; ^3^ Department of Biomedical Sciences Humanitas University Pieve Emanuele Italy; ^4^ Department of Biomedical Sciences for Health University of Milan Milan Italy; ^5^ IRCCS Ospedale Galeazzi—Sant'Ambrogio, Unità Operativa E.U.O.R.R. Milan Italy

**Keywords:** cartilage, mesenchymal stromal cells, orthobiologics, osteoarthritis, soluble factors

## Abstract

**Purpose:**

Orthobiologics gained popularity for the treatment of musculoskeletal pathologies, including osteoarthritis (OA). Bone marrow aspirate concentrate (BMAC) and adipose‐derived stromal vascular fraction (SVF) were reported to reduce OA symptoms, contributing to the restoration of joint homoeostasis. Variability in production protocols and lack of extensive characterisation hinder a clear indication for the choice of a product over the other. The purpose of this study was to characterise side‐by‐side BMAC and SVF obtained with the same family of devices, by assessing cell immunophenotype, release of soluble factors and their ability to reduce inflammation in a pathologic in vitro chondrocyte model.

**Methods:**

BMAC (iliac crest) and SVF (abdomen liposuction) were obtained from 28 (55 years old ± 8) and 39 patients (56 years old ± 9), with Hy‐Tissue BMAC and Hy‐Tissue SVF. BMAC/SVF were characterised for cellular content. The number of mesenchymal stromal cells (MSCs) was investigated by flow cytometry (CD45^−^CD31^−^CD34^+^CD90^+^CD105^−/L^CD146^−^, adipose‐MSCs; CD45^−^CD271^+^, bone marrow‐MSCs). Two‐hundred factors (cytokines, chemokines, receptors, growth factors and inflammatory molecules) were tested by enzyme‐linked immunosorbent assay (ELISA). Anti‐inflammatory potential was assayed in vitro on interleukin‐1 beta (IL1β)‐treated chondrocytes by quantitative reverse transcription polymerase chain reaction (qRT‐PCR) arrays of 84 genes involved in inflammatory processes.

**Results:**

BMAC had higher concentration for white cells (213x), erythrocytes (49x) and platelets (25x), while the number of MSCs resulted comparable between the two products (1000 cells/mL). One‐hundred and twenty‐one soluble factors were identified in all analysed samples, with 88 more abundant in BMAC and one in SVF. Gene ontology revealed that the higher concentrated molecules were mainly growth factors and/or involved in differentiation processes. Both orthobiologics reduced inflammation in the in vitro chondrocyte model, with BMAC showing higher efficacy.

**Conclusions:**

Using specific commercial systems, both orthobiologics showed anti‐inflammatory effects in vitro. BMAC had higher blood cell and growth factor concentrations than SVF, with greater efficacy. However, variability in commercial systems limits generalisation, requiring further study to draw conclusions when different devices are employed.

**Level of Evidence:**

NA

**Abbreviations**
ASC: adipose‐derived MSC
BMA: bone marrow aspirate
BMAC: bone marrow aspirate concentrate
BMSC: bone marrow‐derived MSC
MSC: mesenchymal stromal cell
OA: osteoarthritis
PCA: principal component analysis
PLT: platelets
RBC: red blood cells
SVF: stromal vascular fraction
WBC: white blood cells

## BACKGROUND

Orthobiologics are products prepared at the point of care (POC) through minimal manipulation starting from biological substances and used for the treatment of a variety of musculoskeletal pathologies, including but not limited to chondropathy, osteoarthritis (OA), muscle and tendon disorders. Over the past years, their use gained significant attention due to minimal invasiveness, great healing potential and reasonable cost as a nonoperative treatment option. Particular interest has been devoted to blood‐derived products such as platelet‐rich plasma (PRP) [19], and to cell‐based ones such as those obtained from bone marrow [10], adipose tissue [32] and foetal annexes, the latter for allogenic use only. Autologous cell‐based therapies (CBT) prepared at the POC mainly rely on the presence of mesenchymal stromal cells (MSCs) promoting inflammation reduction and restoration of tissue through the release of growth factors and extracellular vesicles (EVs) embedded with regulatory molecules like microRNAs (miRNAs) [9]. In bone marrow, the percentage of MSCs (BMSCs, bone marrow‐MSCs) is estimated to be between 0.001% and 0.01% of the total mononuclear cells [34] that may range up to 1 × 10^7^/mL [31], therefore with a final expected concentration of 1–10 × 10^4^ MSCs/mL. Several systems to enrich bone marrow aspirate (BMA) in MSCs have been introduced in the market (bone marrow aspirate concentrate, BMAC), which can increase up to fivefold the total number of cells without losing BMSCs. In adipose tissue, the number of MSCs (ASCs, adipose‐MSCs) per gram is estimated to be up to 20–200 times higher with respect to bone marrow [12], with an expected concentration of 0.2–2 × 10^6^ ASCs/g. ASCs lie within the stromal vascular fraction (SVF) that includes all the adipose tissue cells with the exception of adipocytes and can be isolated by either mechanical methods or enzymatic means, with the last ones that are excluded from regulatory‐approved minimal manipulation guidelines [38].

Despite both BMAC and SVF have been extensively investigated for the treatment of OA [3, 6] with satisfactory results, the large variety of clinical settings and different protocols, and the lack of comparative studies with thorough characterisation of the administered products undermine a clear recommendation for the tailored use of BMAC or SVF. Both products present advantages and disadvantages, as well as slight differences in indications. For example, given the inverse correlation between age and cell performances highlighted for BMSCs [11], adipose tissue is preferred to treat older patients. On the other hand, bone marrow‐based cell therapy is more indicated in skinny patients because of the difficulty of harvesting a sufficient amount of adipose tissue from these patients without incurring a too invasive procedure or harvesting fibrotic adipose tissue.

However, besides these empiric observations, the literature lacks a thorough evaluation of the possible differences at both cellular and molecular levels between adipose tissue‐ and bone marrow‐derived CBT prepared at the POC.

The aim of this study was to compare BMAC and SVF, obtained using Hy‐Tissue BMC and Hy‐Tissue SVF (Fidia Farmaceutici S.p.A.), in terms of cellular and soluble factor levels, as well as their paracrine activity on chondrocytes. These two products follow a methodological workflow that includes filtration and centrifugation and is adapted to the optimal processing of the starting tissue, either lipo‐aspirate or bone marrow‐aspirate. The study hypothesis is that SVF and BMAC may exert their effects on different targets and/or in a different extent in this in vitro model. The findings of this study would be useful to reinforce the current knowledge on the use of orthobiologics and possibly drive the choice of a system over the other within this family of devices aimed to be prepared at POC.

## METHODS

### Specimen collection

SVF (from abdomen liposuction) was collected from 39 OA patients (26 males, 13 females; 56 years old ± 9, S1–S39) with Kellgren–Lawrence (KL) II‐III undergoing regenerative medicine injective procedure. BMA (from iliac crest) and BMAC were collected from 28 OA patients (17 males, 11 females; 55 years old ± 8, B1–B28) with KL II‐III undergoing regenerative medicine injective procedure. SVF and BMAC were prepared with the Hy‐Tissue family system, based on subsequent filter and centrifuge steps, following the manufacturer's instructions (Fidia Farmaceutici S.p.A.). A small residual volume remaining after injection into the patient's joint was collected in sterile tubes in the operating room from the syringe after removal of the needle, transported to the research laboratory, and processed within 1 h of collection. All analyses were performed within 2 h of preparation. For all samples, a complete cell content was assessed with a Sysmex XN‐2000 hemocytometer (Sysmex). Depending on the residual volume after cell count, if present, either (i) flow cytometry or (ii) biochemical characterisation or (iii) functional experiment on chondrocytes was performed. When products’ volume after cell count was enough, more than a single analysis was performed on the same sample. This is because obtaining sufficient material for all laboratory analyses while meeting the minimum volume required for patient treatment was not always feasible. This procedure was followed since several factors could impact the final product yield, including the volume of collected tissue, tissue morphology (such as the presence of fibrous fat), and patient hydration, which influences the recovered volume of processed material. Since ensuring the necessary treatment volume was the top priority, we focused on making the most of the available samples. Recognising the value of patient‐derived biological material, we opted not to discard any samples and conducted as many tests as possible with the available volume.

### Flow cytometry

Freshly prepared SVF (*N* = 27) was incubated for 10 min at room temperature (RT) with 10 volumes of red blood cells (RBC) lysis buffer (Miltenyi Biotec) to remove erythrocytes. After centrifugation, the pellet was washed once with 10 mL fluorescence‐activated cell sorting (FACS) buffer and eventually suspended in 200 µL FACS buffer. Aliquots consisting of either unstained (negative control) or stained cells with the combination of the antibodies anti‐CD45‐PE‐Vio770 (REA747, Miltenyi), CD31‐APC (WM59, Biolegend), CD34‐PE (AC136, Miltenyi), CD90‐FITC (REA897, Miltenyi), CD105‐PerCP‐Vio700 (REA794, Miltenyi), CD146‐APC/Fire (P1H12, Biolegend) were prepared as previously reported [34]. Cells were incubated for 30 min at 4°C in the dark, following the manufacturer's instructions. Then, they were washed with 1 mL FACS buffer and the pellet suspended in 250 µL FACS buffer. All samples were acquired using a flow cytometer (Cytoflex, Beckman Coulter Inc.) and analysed with CytExpert software (Beckman Coulter Inc.). ASCs population was identified as CD45^−^CD31^−^CD34^+^CD90^+^CD105^−/L^CD146^−^. The ASCs number per millilitre was calculated as % of ASCs events on CD45^+^ events and this ratio was multiplied by the white blood cells (WBC) count obtained with the hemocytometer.

Freshly prepared BMA and BMAC (*N* = 23) were treated with RBC lysis buffer and washed as previously described, and then suspended in 200 μL FACS buffer. One aliquot of cells was stained with anti‐CD45‐PE‐Vio770 and CD271‐PE (345105, Biolegend) antibodies for 30 min at 4°C in the dark, while the remaining cells were maintained unstained as a control [44]. After one wash in FACS buffer, the samples were suspended in 1000 µL FACS buffer and data were acquired by flow cytometry and analysed with CytExpert software. BMSC population was identified as CD45^‐^CD271^+^. The BMSCs number per millilitre was calculated as % of BMSCs events on CD45^+^ events and this ratio was multiplied for the WBC count obtained with the hemocytometer.

### Soluble factors quantification

Before quantification, fresh SVF (*N* = 12) and BMAC (*N* = 8) were processed to remove the cellular components. SVF was centrifuged for 400*g* for 5 min and 2000*g* for 10 min before the supernatant was filtered with a 0.22 µm device. BMAC was 1:1 diluted in saline solution and centrifuged at 4000*g* for 5 min. Samples were kept frozen at –80°C until use. Two hundred fifty microliters of processed SVF or BMAC were twofold diluted before secreted factors detection with the enzyme‐linked immunosorbent assay (ELISA) Quantibody® Human Cytokine Array 4000 Kit (RayBiotech, https://www.raybiotech.com/quantibody-human-cytokine-array-4000/) following the manufacturer's protocol and four technical replicates. Concentrations were determined by comparison with standard samples taking into account sample dilution. Only factors detected above threshold sensitivity for each single assay in all analysed samples were further processed. Values are reported as pg/mL.

### Protein–protein interaction network analysis

Interactome maps of ELISA‐identified proteins were generated with the STRING tool [41] (http://www.string-db.org) (database v12.0). The following settings were selected: (i) organism: Homo sapiens; (ii) network type: full STRING network with the edges indicating both functional and physical protein associations; (iii) meaning of network edges: evidence with line colour indicating the type of interaction evidence; (iv) sources for active interaction evidence: curated databases; (v) minimum required interaction scores: medium confidence (0.400, default value). This score does not indicate the strength or the specificity of the interaction, but it is an indicator of confidence, that is, how likely STRING judges an interaction to be true, given the available evidence. All scores rank from 0 to 1, with 1 being the highest possible confidence. A score of 0.5 would indicate that roughly every second interaction might be erroneous (e.g., a false positive).

### Functional experiment on chondrocytes

Immortalised chondrocytes (InSCREENex GmbH; PrNa1K21; P5) were seeded at 90,000 cells/cm^2^ in DMEM/F12 medium (Thermo Fisher Scientific) supplemented with 10% foetal bovine serum (FBS) (Thermo Fisher Scientific) in 24‐wells plates. After 8 h to allow cell adhesion, the medium was discarded, and wells were washed with phosphate‐buffered saline (PBS). By means of transwell system, the following conditions were set: (i) 300 µL saline solution (CTRL); (ii) 300 µL saline solution with 1 ng/mL interleukin‐1 beta (IL‐1β) (Sino Biological) added in the lower chamber (900 µL in the lower chamber + 300 µL in the upper chamber) (IL‐1β); (iii) 300 µL fresh SVF with 1 ng/mL IL‐1β added in the lower chamber (900 µL in the lower chamber + 300 µL in the upper chamber) (IL1B + SVF); (iv) 300 µL fresh BMAC with 1 ng/mL IL‐1β added in the lower chamber (900 µL in the lower chamber + 300 µL in the upper chamber) (IL1B + BMAC). After 48 h, chondrocytes were collected and RNA extracted with RNeasy Kit (Qiagen). Reverse transcription was performed on 100 ng RNA by using the RT^2^ First Strand Kit (Qiagen). The complementary DNA (cDNA) was pre‐amplified with the RT^2^ PreAMP cDNA Synthesis Kit (Qiagen) and amplification performed on the real‐time RT² Profiler™ PCR Array Human Cytokines & Chemokines (QIAGEN, Cat. no. PAHS‐150ZE) in combination with RT² SYBR Green qPCR Mastermix (Qiagen). Each array plate contained four sets of 96 wells for testing. Genomic DNA contamination, reverse transcription and positive PCR controls were included in each 96‐well set. After housekeeping gene (HKG) stability testing, *RPLP0* was used as the assay reference gene after selection for its stability as reported below. Values are reported as fold versus CTRL set as 1. Altogether, due to single or multiple orthobiologics availability from the surgery room in the same day, seven independent experiments on chondrocytes were performed for a total of 10 SVF and 10 BMAC.

### Analysis of housekeeping gene stability

Four algorithms were used to test HKG stability: comparative Δ*C*_t_ method [39], BestKeeper [33], NormFinder [2] and geNorm [43]. Each algorithm evaluates HKG reliability with different variables. In the Δ*C*_t_ approach, ‘pairs of genes’ are compared. BestKeeper uses standard deviation (SD), with low stability marked by a higher value. Normfinder allows the definition of a stability value (high stability for low value). An M‐value based on the average pairwise expression ratio is given by geNorm, with stability being defined by M < 1.5. Eventually, a stability ranking is provided by each approach generated. The four rankings were computed by RefFinder [46], which calculates the geometric mean of the different rankings to generate the overall final ranking.

### Clustering analyses

Principal component analysis (PCA) and hierarchical clustering were conducted with the ClustVis package [30] (https://biit.cs.ut.ee/clustvis/). Maps were generated using the following settings. (i) soluble factors: ln transformation of pg/mL values; no row centreing; no row scaling; PCA method: SVD with imputation; clustering distance for rows and columns, correlation; clustering method for rows and columns, average; tree ordering for rows and columns, tightest cluster first. (ii) gene expression: as above with the difference of using *RPLP0*‐normalised *C*_t_ values.

### Statistical analyses

For gender distribution between the BMA/BMAC and SVF donors, the *χ* ^2^ test of independence was used with a significance level set at 0.05. For direct comparison of BMA and BMAC parameters, a Shapiro–Wilk test for normal distribution was performed (*α* = 0.01). When passed, a paired *t* test was used, otherwise a Wilcoxon matched‐pairs signed rank test was used. For direct comparison of SVF and BMAC parameters, a Shapiro–Wilk test for normal distribution was performed (*α* = 0.01). When passed, an unpaired *t* test was used, otherwise a Mann–Whitney test was used. For gene expression comparing multiple conditions, a Shapiro–Wilk test for normal distribution was performed (*α* = 0.01). With normal values, an ordinary one‐way analysis of variance (ANOVA) was performed with Tukey correction for multiple comparisons, otherwise a Kruskal–Wallis test was done with Dunn's test for multiple comparisons. Significance was set for *p* value ≤ 0.05 couple with a fold change ≤ 0.5 or ≥ 2. Data are presented as mean ± SD. For correlation analysis, blood cell counts (×10^3^/µL), the concentration of soluble factors (pg/mL) and the modulation of genes in inflamed chondrocytes expressed as fold‐change versus IL1β samples were computed. For normally distributed data in both data sets to be correlated (Shapiro–Wilk test for normal distribution, *α* = 0.01) the Pearson correlation coefficient is shown. For not‐normally distributed data in at least one of the two data sets to be compared, the Spearman correlation was performed. Correlation (positive or negative) was reported only when very strong [1], namely when ≤ –0.8 or ≥ 0.8 with a *p* value ≤ 0.05. Analyses were performed with GraphPad Prism v8.0.2 (GraphPad Software).

## RESULTS

### SVF and BMAC characterisation

**Figure 1:**
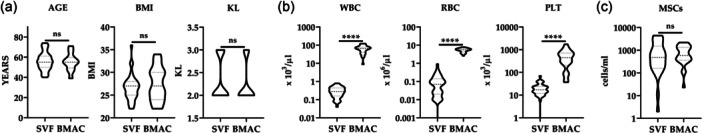
Orthobiologics characterisation. (a) Stromal vascular fraction (SVF) and bone marrow aspirate concentrate (BMAC) donor age, body mass index (BMI) and Kellgren–Lawrence (KL). Violin plots with quartiles and median are shown. *N* = 39 for SVF and 28 for BMAC. ns stands for not significant. (b) Haematological data for SVF and BMAC. Violin plots with quartiles and median are shown. *N* = 39 for SVF and 27 for BMAC. **** for *p* value < 0.0001. (c) Mesenchymal stromal cells (MSCs) content per millilitre in SVF and BMAC. Violin plots with quartiles and median are shown. *N* = 27 for SVF and 23 for BMAC. ns stands for not significant.

Age of SVF and BMA/BMAC donors did not show statistical difference, being 56 years old ± 9 and 55 years old ± 8, respectively (Figure 1a), as well as body mass index (BMI), being 27 ± 3 and 27 ± 4 (Figure 1a), and KL, being 2.3 ± 0.5 and 2.3 ± 0.4 (Figure 1a). In both groups, men were more represented (M/F ratio of 2.0 and 1.5 in SVF and BMA/BMAC, respectively, with no statistical difference for gender distribution for the two products). Complete data of all the samples are summarised in Supporting Information S2: Table S1. Mean WBC, RBC and platelets (PLT) count for SVF, BMA and BMAC are reported in Table 1. Bone marrow aspirate concentration (BMAC vs. BMA) led to a significant (*p* < 0.0001) increase in all counts, with higher incremental values for WBC (6.0‐fold) and PLT (4.5), while RBC had a lower accumulation (1.4). Also, as expected, all BMAC counts were significantly higher (*p* < 0.0001) than SVF ones: WBC (213‐fold), RBC (49) and PLT (25) (Figure 1b).

**Table 1:** Mean WBC, RBC and PLT count for SVF, BMA and BMAC.

	WBC	RBC	PLT
SVF	0.30 × 10^3^/µL ± 0.18	0.11 × 10^6^/µL ± 0.16	20 × 10^3^/µL ± 13
BMA	10.48 × 10^3^/µL ± 3.81	3.75 × 10^6^/µL ± 0.52	114 × 10^3^/µL ± 50
BMAC	64.10 × 10^3^/µL ± 28.66****^/§§§§^	5.33 × 10^6^/µL ± 1.31****^/§§§§^	506 × 10^3^/µL ± 403****^/§§§§^

*Note*: *N* = 39 for SVF, *N* = 27 for BMA and *N* = 27 for BMAC. **** for *p* value < 0.0001 for BMAC versus BMA and ^§§§§^ for *p* value < 0.0001 for BMAC versus SVF.

Abbreviations: BMA, bone marrow aspirate; BMAC, bone marrow aspirate concentrate; PLT, platelets; RBC, red blood cells; SVF, stromal vascular fraction; WBC, white blood cells.

Flow cytometry was used to identify the MSCs population in both groups. In SVF, ASCs were defined by the signature CD45^−^CD31^−^CD34^+^CD90^+^CD105^−/L^CD146^−^ (Supporting Information S1: Figure S1A). With respect to CD45^+^ cells, ASCs resulted 0.67% ± 1.13 for an amount of 1093 ± 1335 cells/mL. In BMA and BMAC, BMSCs were scored by: CD45^‐^CD271^+^ (Supporting Information S1: Figure S1B). With respect to CD45^+^ cells, BMSCs in BMA were 0.0011% ± 0.0007 and in BMAC were 0.0013% ± 0.0009 with no significant difference. Due to total cell concentration in BMAC, the BMSCs count per millilitre had a sevenfold increase, increasing from 126 ± 81 (BMA) to 859 ± 649. Of note, no significant difference in MSCs/mL emerged between SVF and BMAC (Figure 1c).

### Soluble factors

**Figure 2:**
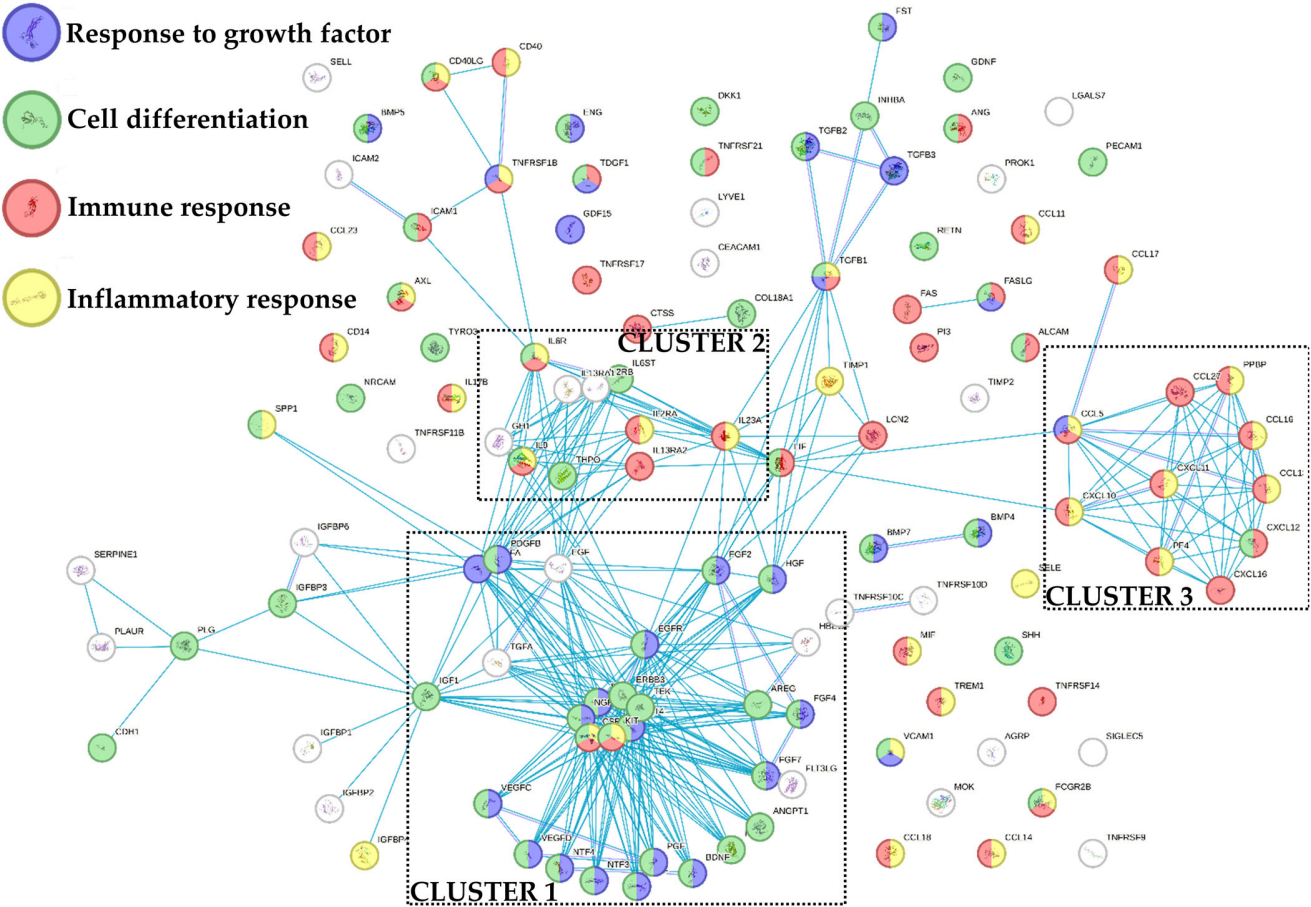
Functional association network for identified bone marrow aspirate concentrate (BMAC) and stromal vascular fraction (SVF) soluble factors. Blue connections are for proteins with known interactions based on curated databases. Empty nodes, proteins of unknown three‐dimensional (3D) structure; filled nodes, known or predicted 3D structure.

One hundred and twenty‐one soluble factors were detected in all samples (Supporting Information S3: Table S2). To identify the most relevant functions for these molecules, a protein association network analysis was performed sifting only the interactions from curated databases (Figure 2). Three main clusters emerged. The most relevant one (Cluster 1) was composed of several proteins mainly related to ‘Response to growth factor’ and ‘Cell differentiation’ biological processes terms (GO:0070848 and GO:0030154, respectively). Accordingly, growth factors and their receptors laid in this group. Those proteins were connected to a smaller and looser cluster (Cluster 2) based on interleukins and their receptors, related to the abovementioned GO terms and to ‘Inflammatory response’ and ‘Immune response’ ones (GO:0006954 and GO:0006955, respectively). These two GO terms sharply defined the last cluster (Cluster 3), composed of chemokines with C–C and C–X–C motifs. Eventually, transforming growth factor beta (TGFB) members (1/2/3) defined a small group connected with Clusters 1 and 2.

**Figure 3:**
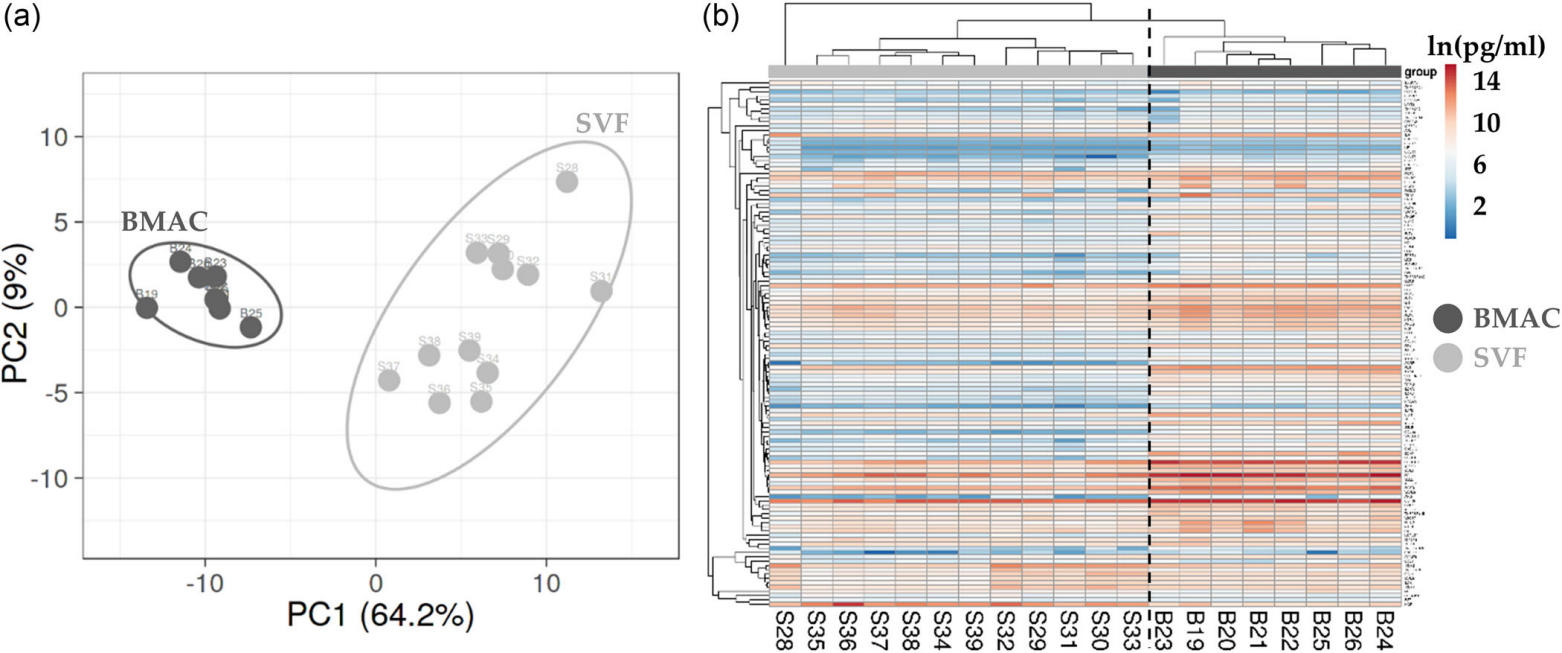
Soluble factors profile comparison between bone marrow aspirate concentrate (BMAC) and atromal vascular fraction (SVF) samples. (a) Principal component analysis of the ln(x)‐transformed pg/million values of detected factors. X and Y‐axis show principal component 1 and principal component 2, which explain 64.2% and 9% of the total variance. (b) Heat map of hierarchical clustering analysis of the ln(x)‐transformed pg/million values of detected factors with sample clustering tree at the top and factors clustering tree on the left side.

When comparing data sets, PCA and hierarchical clustering analyses revealed a clear and distinct separation (Figure 3). On average, BMAC samples had a 4.8‐fold higher concentration for detected soluble factors. For BMAC, two factors (KIT and CSF1R) had an amount ≥ 1000 ng/mL, 4 (PDGFA, EGFR, BMP5 and PLG) ≥ 100 ng/mL, 38 ≥ 10 ng/mL, 39 ≥ 1 ng/mL and 39 < 1 ng/mL. For SVF, three proteins (CSF1R, hepatocyte growth factor [HGF] and KIT) were ≥ 100 ng/mL, 18 ≥ 10 ng/mL, 41 ≥ 1 ng/mL and 60 < 1 ng/mL. Table 2a,b shows the top 10 factors for both orthobiologics. Regarding the differential amount, 12 proteins had a significant (*p* ≤ 0.05) BMAC/SVF fold ≥ 10, 17 ≥ 5, 60 ≥ 2 and only one was more concentrated in SVF (HGF, BMAC/SVF of 0.1). In Table 2c, the top 10 enriched factors in BMAC are shown.

**Table 2:** Top enriched soluble factors in BMAC and SVF.

(a)	BMAC		(b)	SVF		(c)	BMAC/SVF	
Factor	ng/mL	SD	Factor	ng/mL	SD	Factor	Fold	*p* value
KIT	1255	525	CSF1R	335	119	CCL16	43.7	<0.0001
CSF1R	1098	328	HGF	196	232	BDNF	39.2	<0.0001
PDGFA	725	246	KIT	178	123	PDGFA	32.7	<0.0001
EGFR	299	81	BMP5	73	35	IGFBP3	14.2	<0.0001
BMP5	174	65	TIMP2	67	56	AGRP	13.7	<0.0001
PLG	109	32	PDGFA	65	38	SERPINE1	13.6	<0.0001
FGF4	98	35	EGFR	53	20	ERBB3	13.5	0.0018
PROK1	72	32	FGF2	38	17	KITLG	11.3	0.0015
VCAM1	72	33	FGF4	33	13	PDGFA	11.2	<0.0001
IL‐9	67	22	IL‐9	32	26	CCL23	10.6	<0.0001

Abbreviations: BMAC, bone marrow aspirate concentrate; HGF, hepatocyte growth factor; SVF, stromal vascular fraction.

**Figure 4:**
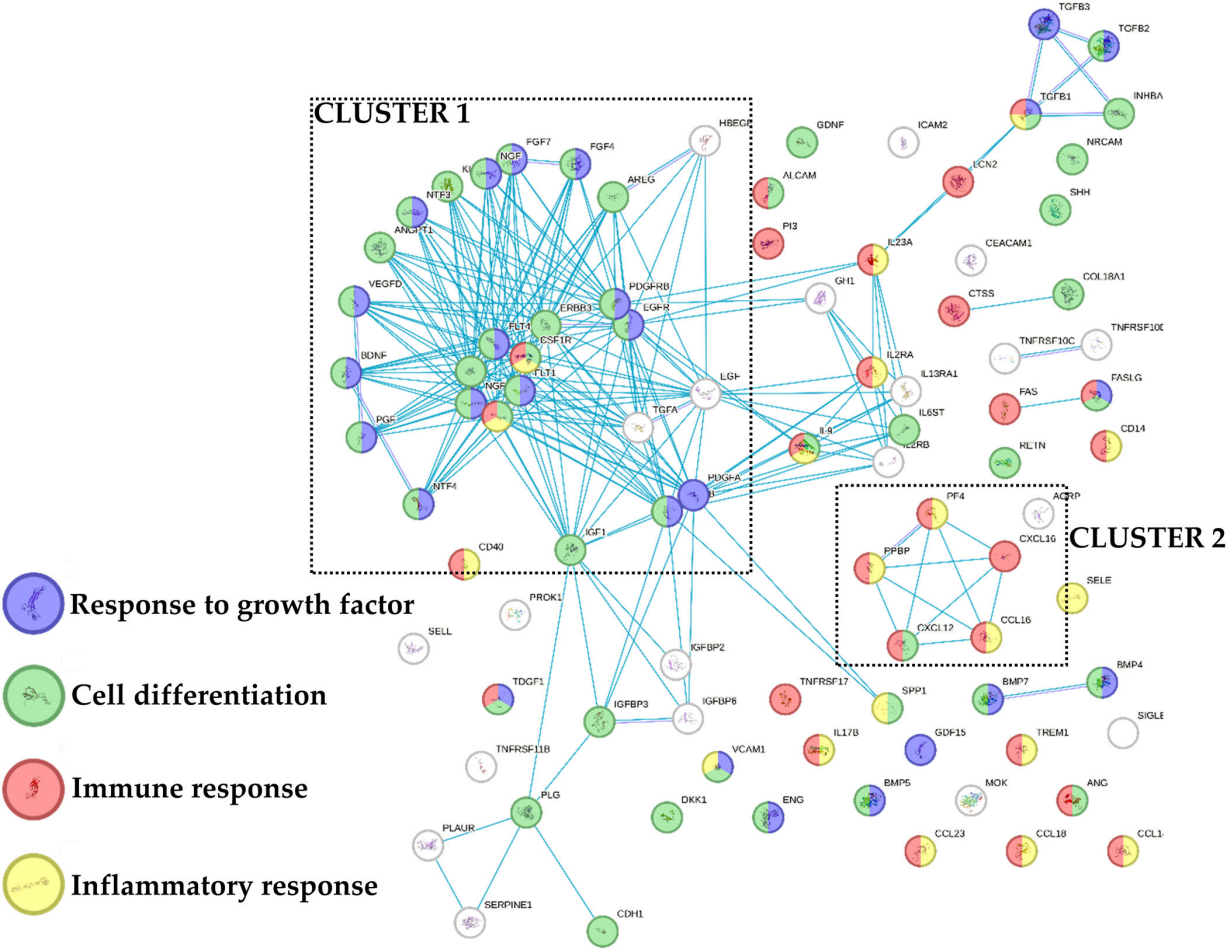
Functional association network for proteins that resulted more concentrated in bone marrow aspirate concentrate (BMAC). Blue connections are for proteins with known interactions based on curated databases. Empty nodes, proteins of unknown three‐dimensional (3D) structure; filled nodes, known or predicted 3D structure.

Eventually, the 89 proteins that resulted more concentrated in BMAC were tested by functional annotation analysis (Figure 4). Of note, with respect to the previously identified 4 GO terms, ‘Response to growth factor’ and ‘Cell differentiation’ described the main cluster (Cluster 1) while ‘Inflammatory response’ and ‘Immune response’ were associated with less connected factors with the exception of a C–C and C–X–C motifs chemokines‐based cluster (Cluster 2). A further analysis revealed the presence of five proteins related to ‘Cartilage development’, namely BMP4/5/7, TGFB1 and FGF4 (GO:0051216). Also, sifting for orthopaedic conditions, 20 factors were related to ‘Musculoskeletal system disease’ [DOID:17], mainly at inflammatory (‘Inflammatory’ and ‘Immune response’: KIT, CSF1R, CD40, IL2RA, IL23A, TGFB1 and FAS) and matrix (‘positive regulation of collagen biosynthetic process’, GO:0032967: TGFB1, BMP4 and PDGFRB) levels. Thus, differentially abundant proteins might influence both homoeostasis and inflammatory levels of target tissues as cartilage.

### Analysis of housekeeping genes stability for molecular analyses

The reliability of five common HKGs was scored in inflamed chondrocytes treated with the two orthobiologics under study. Four applets were used, and the eventual stability ranking was generated (Table 3). *RPLP0* resulted the most stable candidate (geomean of the four applets’ rankings = 1.32), followed by *B2M* (1.68) and *ACTB* (2.28). *GAPDH* and *HPRT1* laid in fourth and last position, respectively, with all algorithms. Thus, *RPLP0* was used as HKG for further analyses.

**Table 3:** Stability rankings of tested HKGs for inflamed chondrocytes treated with BMAC or SVF.

RANKING	GEOMEAN		DELTA CT		BESTKEEPER		NORMFINDER		GENORM	
1	*RPLP0*	1.32	*RPLP0*	0.69	*RPLP0*	0.19	*RPLP0*	0.32	*ACTB* | *B2M*	0.53
2	*B2M*	1.68	*B2M*	0.71	*B2M*	0.44	*B2M*	0.40		
3	*ACTB*	2.28	*ACTB*	0.72	*ACTB*	0.48	*ACTB*	0.42	*RPLP0*	0.56
4	*GAPDH*	4.00	*GAPDH*	0.86	*GAPDH*	0.66	*GAPDH*	0.68	*GAPDH*	0.66
5	*HPRT1*	5.00	*HPRT1*	1.01	*HPRT1*	0.71	*HPRT1*	0.90	*HPRT1*	0.80

*Note*: GeNorm indicates the most stable couple, thus second position of the ranking is missing.

Abbreviations: BMAC, bone marrow aspirate concentrate; SVF, stromal vascular fraction.

### BMAC and SVF effect on inflamed chondrocytes

**Figure 5:**
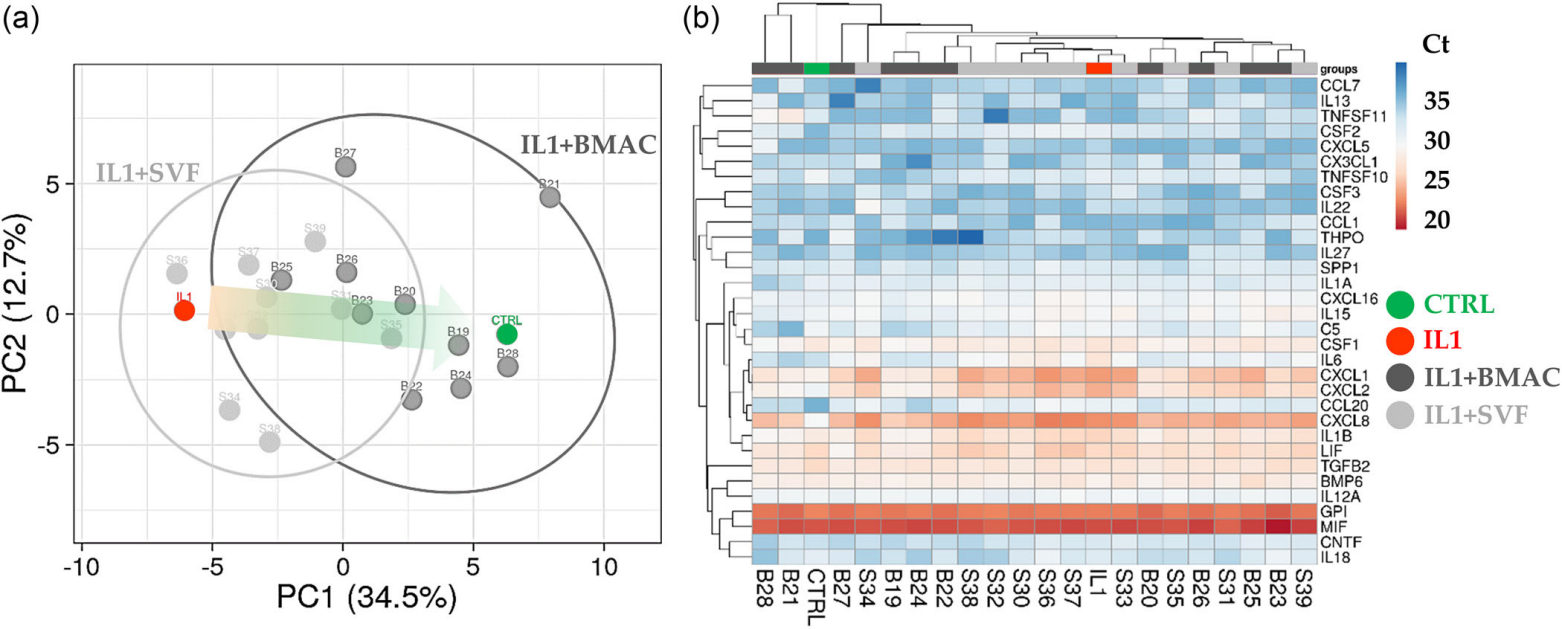
Inflammation‐related gene expression comparison profile for Interleukin‐1 beta (IL1β)‐chondrocytes treated with bone marrow aspirate concentrate (BMAC) or stromal vascular fraction (SVF). (a) Principal component analysis of the *RPLP0*‐normalised *C*_t_ values of detected genes. X and Y axes show principal component 1 and principal component 2, which explain 34.5% and 12.7% of the total variance. (b) Heat map of hierarchical clustering analysis of the *RPLP0*‐normalised *C*_t_ values of detected genes with sample clustering tree at the top and gene clustering tree on the left side. For both A and B panels, CTRL and IL1β‐treated chondrocytes mean values are reported for ease of Figure readability.

Out of 84 tested genes, 32 were detected in all analysed samples (Supporting Information S4: Table S3). At a global level, PCA clearly showed that IL1β was able to strongly impact the gene expression signature of chondrocytes and that SVF and BMAC were able to counteract the transcriptional trend given by inflammation (Figure 5a). Of note, it was not possible to obtain a sharp dichotomy between the two orthobiologics, as highlighted by hierarchical clustering (Figure 5b), suggesting an overlapping behaviour. To get into more details in the gene regulation given by BMAC and SVF, a focused analysis was performed on those genes specifically altered by IL1β.

**Figure 6:**
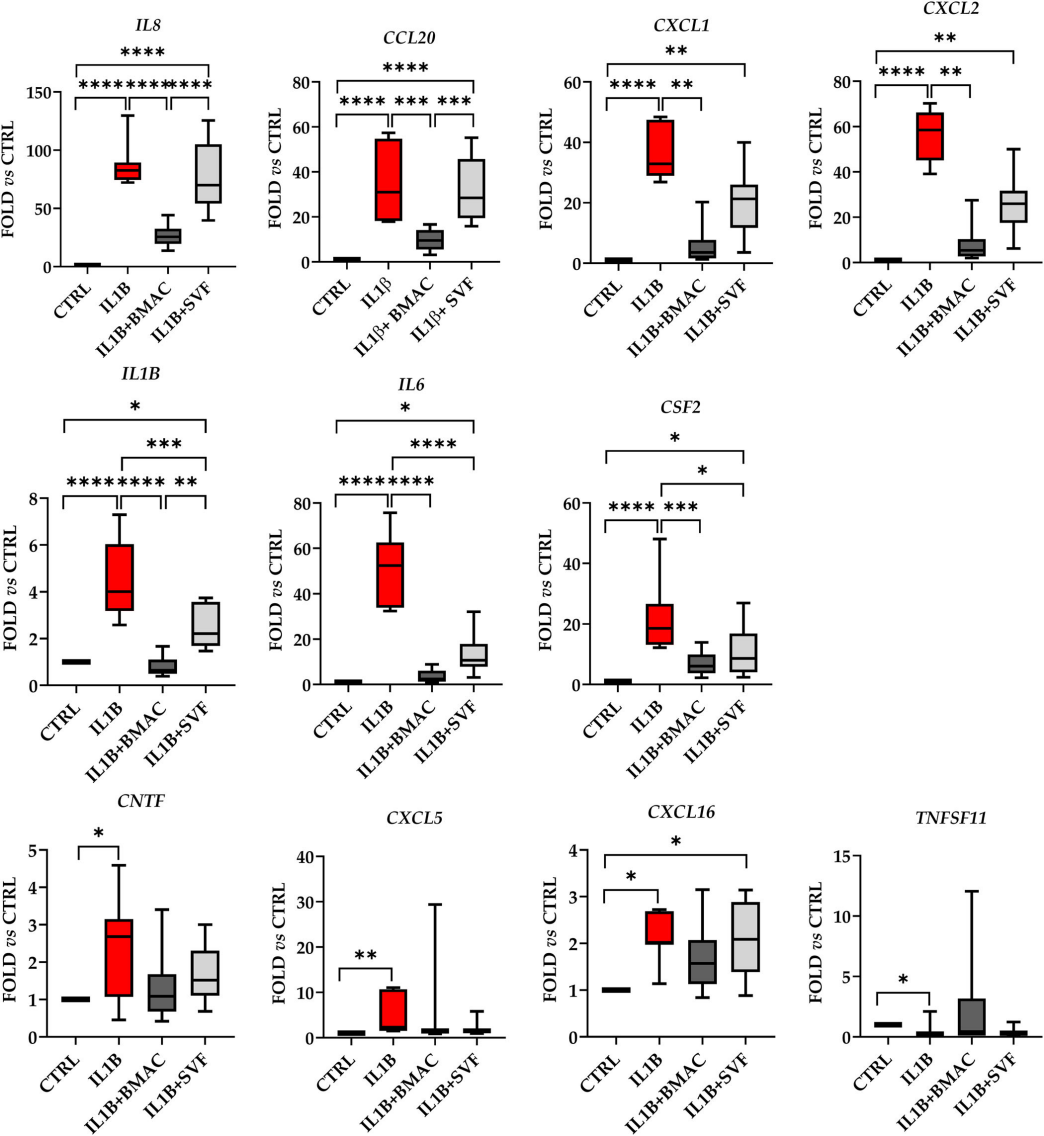
Gene expression modulation in inflamed chondrocytes by bone marrow aspirate concentrate (BMAC) or stromal vascular fraction (SVF) treatment. Interleukin‐1 β (IL1β)‐inflamed (IL1B) chondrocytes treated with BMAC (IL1B + BMAC) or SVF (IL1B + SVF) were analysed for the expression of genes involved in inflammation and their amount compared to untreated (CTRL) cells set as 1. Only significantly (fold ≥ 2 or ≤ 0.5 and *p* value ≤ 0.05) modulated genes are shown. * for *p* value ≤ 0.05, **≤ 0.01, ***≤ 0.001 and ****≤ 0.0001. *N* = 7 for CTRL and IL1B, *N* = 10 for IL1B + BMAC and IL1B + SVF. Box and whiskers are shown with min to max values.

Inflammation was able to significantly (fold change ≥ 2 or ≤ 0.5 with *p* value ≤ 0.05) upregulate the expression of 10 genes and downregulate the amount of one candidate (Figure 6). For the increased transcripts, five encode chemokines (*CXCL1/2/5/16* and *CCL20*), three interleukins (*IL1*/6/8) and two growth factors (*CNTF* and *CSF2*). The downregulated gene is coding for a receptor of tumour necrosis factor α (TNFα) (*TNFSFS11*). BMAC was able to specifically downregulate the expression of *IL8* and *CCL20*, which were not changed in their amount by SVF. Other five genes were reduced by both orthobiologics with different patterns. For *CXCL1/2*, the high heterogeneity in SVF samples did not allow to reach statistical significance observed for BMAC. *IL1β* had a similar trend, with SVF‐treatment allowing for significant reduction albeit weaker than BMAC. *IL6* and *CSF2* showed a close downregulation for both treatments, with no difference between SVF and BMAC. Of note, for all these genes, BMAC values never showed a significant difference with respect to control unlike SVF samples where reduction was not completely reached. Eventually, four genes (*CNTF*, *CXCL5/16* and *TNFSF11*) had very high variations between samples making statistical analysis less effective. Nevertheless, the observed modulation by IL1β treatment was lost with both BMAC and SVF with the only exception of *CXCL16* where upregulation upon inflammation remained.

### Correlation analysis

**Figure 7:**
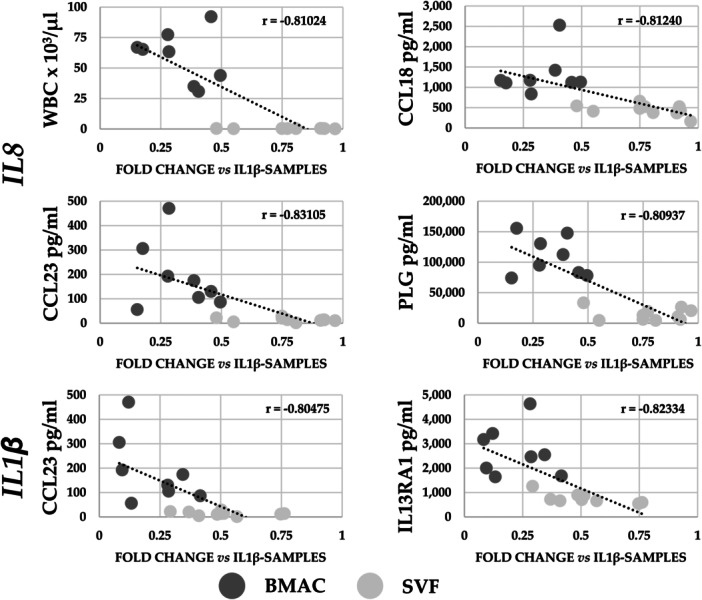
Correlation analysis of bone marrow aspirate concentrate (BMAC) and stromal vascular fraction (SVF) parameters with modulation in gene expression in inflamed chondrocytes. Correlations with ‘*r*’ values being ≤–0.8 or ≥0.8 with *p* value ≤ 0.05 are presented. *N* = 8 for BMAC and *N* = 10 for SVF.

A correlation analysis encompassing all significantly different values between BMAC and SVF was performed for samples obtained from the same donors (*N* = 8 for BMAC and *N* = 10 for SVF). Computed parameters were: WBC, RBC and PLT, alongside the 89 soluble proteins with differential amount and the seven genes with the most effective modulation in inflamed chondrocytes (*CXCL1/2*, *CCL20*, *CSF2*, *IL1/6/8*). The analysis was focused on the downregulation in gene expression (fold change of IL1β + BMAC or IL1β + SVF vs. IL1β) and the correlation with blood counts or factors’ concentration. The reduction in the expression of two genes resulted correlated with the following parameters (Figure 7): *IL8* downregulation had a negative correlation with WBC (‘*r*’ of –0.81), CCL23 (–0.83), CCL18 (–0.81) and PLG (–0.81) concentration; *IL1β* reduction was negatively correlated with CCL23 (–0.80) and IL13RA1 (–0.82). These results were corroborated at the single sample level where a sharp dichotomy between BMAC‐ or SVF‐treated samples emerged depending on cell count or factor concentration (Figure 7). Eventually, the correlation analysis showed that differentially expressed genes have a similar trend in the analysed samples with the exception of *CSF2* that had low *r* values (Table 4).

**Table 4:** Correlation analysis for differentially expressed genes in inflamed chondrocytes treated with BMAC or SVF *vs* IL1β.

	*CSF2*	*CXCL1*	*CXCL2*	*IL1β*	*IL6*	*IL8*
*CCL20*	0.11531	**0.82957**	**0.83261**	**0.80863**	0.57667	0.74584
*CSF2*		0.11163	0.02584	0.02120	0.06929	0.16451
*CXCL1*			**0.98596**	0.76882	**0.86014**	**0.88498**
*CXCL2*				0.73676	**0.85522**	**0.85306**
*IL1B*					0.60421	**0.90326**
*IL6*						0.79779

*Note*: ‘*r*’ correlation values with *p* values ≤ 0.05 are shown in bold.

## DISCUSSION

The main finding of this work directly comparing BMAC and SVF samples obtained with specific commercial systems allowed to observe that blood cell count and several soluble factors were more abundant in BMAC, although MSCs content was similar. Both orthobiologics had an anti‐inflammatory effect on inflamed chondrocytes in vitro, with BMAC showing a more pronounced effect. A lower amount of WBC, RBC and PLT was found in SVF. This is consistent with previous works, which characterised SVF produced with the same device used in this study (Hy‐Tissue SVF), where a comparable cell yield in the order of 10^4^–10^5^/mL of processed fat was reported [8, 35]. Also, a similar number of stromal cells was detected (10^2^–10^3^/mL fat), measured as MSCs or able to form CFU [8, 35], a parameter often used to identify MSCs. The presence of this population was defined using flow cytometry, confirming previous findings with different marker panels [8, 35]. Regarding BMAC obtained with Hy‐Tissue BMC, due to its recent release, no reports are available. Of note, the number of MSCs was similar in the two products under study. Considering the higher concentration of MSCs in native adipose tissue than in bone marrow (20–200X), these data underline the higher efficiency of the system for BMAC preparation in retrieving and concentrating MSCs compared with that for SVF. This can be explained by the composition of the adipose tissue, where the presence of a dense ECM makes it difficult to achieve a complete release of cells from the tissue matrix by mechanical process alone, resulting in an inability to recover all MSCs present. This is, however, the result of the compromise necessary to meet the current regulations governing the preparation and use of cell‐based products obtained with minimal manipulation, where enzymatic digestion is in fact not permitted. These data may be due to the combination of the higher cell content in BMAC [31], with low MSCs percentage [34], which is even increased with the concentration procedure from BMA and the reduced cell content in SVF, with high MSCs percentage [12]. The comparable value of approximately 1000 MSCs per millilitre or gram of tissue suggests that the variations between the two orthobiologics in terms of biologic effects highlighted in this study may be ascribed to either different activity of BMSCs and ASCs or the other components present with different amounts such as immune cells, platelets or soluble factors. The higher chondrogenic signature of BMSCs [29] and the greater immunomodulatory ability of ASCs [26] would support the first scenario. Nevertheless, and not necessarily in contrast, an increased amount of blood cells, as in BMAC, might influence orthobiologics potential. The presence of RBC and their by‐products was shown to reduce orthobiologics efficacy affecting resident MSCs [16], synoviocytes [7] and chondrocytes [20] health, together with activating proinflammatory processes in musculoskeletal regenerative therapies [17]. On the contrary, PLT are endorsed with strong regenerative and anti‐inflammatory properties through the release of several growth factors/cytokines [21] and are the basis for the therapies based on PRP [27]. Regarding WBC, there is a debate on their advantage or disadvantage. This is clearly shown in PRP itself, where the WBC content can influence the concentration of various cytokines and modulate local immune and inflammatory responses with both negative and positive effects of leucocyte‐rich PRP on tissue healing reported in the literature [13]. In fact, leucocytes may both trigger catabolic effects through the release of proinflammatory cytokines [25] and positively modulate the immune responses through the secretion of growth factors [40]. Consistently, although some preclinical [7] and clinical evidence [18] have shown a possible local effect of leucocytes able to trigger inflammation, recent high‐level clinical studies have demonstrated no clinical influence of leucocytes on PRP action [15, 37].

In this scenario, in our samples, along a higher number of WBC, also an increased concentration of few cytokines and several growth factors was detected in BMAC with respect to SVF, with the detection of some growth factors (*e.g*., HGF, IGF1, VEGFA and PDGFB), although at a low level, confirming previous data for SVF produced with the same system [14, 23, 36]. By exerting influence over inflammation, growth factors can modulate the microenvironment of chondrocytes to provide an enhanced cartilage regeneration. For instance, growth factors such as PDGFs, TGFβ, FGF2 or IGF1 may interfere with inflammation by blocking the associated signal pathways and promote cartilage regeneration. As an example, PDGFs and TGFβ decrease IL1β‐induced NF‐κB activation, a major pathway involved in the pathogenesis of OA [42]. Of note, in BMAC samples, all these factors, with the exception of FGF2, were more abundant than in SVF, including members of the TGF family (TGFB1/2/3 and TGFA), PDGFA/B, IGF1 and IGF‐binding proteins (IGFBP2/3/6) that are involved in IGF1 stabilisation [4]. Nevertheless, HGF, which also disrupts nuclear factor kappa B (NF‐κB)‐transactivating activity by enhancing cellular nuclear factor of kappa light polypeptide gene enhancer in B‐cells inhibitor alpha expression in chondrocytes [5], was more abundant in SVF suggesting a complex scenario behind orthobiologics differential activity that also involves the presence of soluble receptors. Accordingly, several TNFα receptors were detected, including TNFRSF10C/10D/11B/17, which were more abundant in BMAC together with interleukin receptors IL2RA/B, IL6ST and IL13RA1. In consequence, the in vitro experiment on IL1β‐treated chondrocytes showed for both orthobiologics the ability to reduce inflammation, enhanced for BMAC. Interestingly, the correlation analysis emphasised how this anti‐inflammatory ability was not strictly correlated with the concentration of anti‐inflammatory growth factors or cytokine receptors. In fact, if for WBC an anti‐inflammatory role might be postulated as previously discussed, CCL18/23 are involved in recruiting T lymphocytes and monocytes [22] with CCL18 levels correlated with radiographic grading of knee OA [49]. Notably, CCL23 was reported to reduce *IL8* expression in epithelial cells [28]. IL13RA1 is the receptor of IL13, an anti‐inflammatory cytokine with a protective role in OA [45] that upon binding with its soluble receptor may prolong its function. To be noted that IL13 counter regulates IL1β synthesis [48] and enhances the production of the chondroprotective metalloproteinase ADAM15 in cartilage [48]. At last, PLG in its active form, plasmin, degrades cartilage ECM and activates other metalloproteases (MMPs) contributing to OA [47]. As a matter for future research, another piece of the puzzle explaining the differential anti‐inflammatory potential of SVF might be attributed to the donors’ tendency toward overweight (mean BMI of 27), which is linked to adipose tissue, and stepwise its products, such as SVF, inflammation associated with obesity [24].

All of this suggests that, while from a clinical point of view the use of orthobiological POC products based on the presence of MSCs, such as BMAC and SVF, is easier than the use of culture‐expanded MSCs preparations, the interpretation of the actual effectors of their efficacy is significantly more difficult given the presence of many more players than in MSC cultures alone. Thus, a clear picture to correlate BMAC or SVF composition with their activity on chondrocytes and OA needs further investigation being aware of the wider array of molecules and the potentially different interaction with the whole pathologic joint environment. Likewise, clinical studies to assess the efficacy and the possible differences between SVF and BMAC in treating OA patients are needed to provide clearer indications.

This study has some limitations. The analysis was restricted to well‐known soluble proteins, limiting the scope of tested molecules. A more comprehensive evaluation is needed, including emerging factors like EVs and shuttled miRNAs, which play both therapeutic and pathological roles. In addition, the in vitro model used to test BMAC or SVF activity is a very simplified version of OA. Although unanimously recognised as a good in vitro model of OA for this kind of studies, inflamed chondrocytes are obviously far from the pathologic joint environment that implies complex interaction among other players than cartilage such as synovium, synovial fluid and immune cells. Eventually, the next step of our research will be devoted to evaluating BMAC and SVF properties on OA patients, in a large clinical trial that is ongoing, and identify possible correlations between orthobiologics composition and outcomes.

## CONCLUSIONS

Both BMAC and SVF obtained using the specific commercial systems for their preparation (Hy‐Tissue BMAC and Hy‐Tissue SVF, respectively) demonstrated anti‐inflammatory effects in an in vitro model of pathological chondrocytes, with BMAC showing a higher reduction of IL1β‐induced inflammation, along with greater efficacy. Despite a comparable number of MSCs, the two orthobiologics exhibited distinct cellular and molecular profiles. BMAC also had higher concentrations of blood cells and growth factors compared with SVF. Although these observed differences provide an initial insight into understanding BMAC and SVF outcomes, particularly at the cartilage level in OA patients, the variability in commercial systems used to prepare orthobiologics suggests that these results cannot be generalised. Further investigations are necessary to evaluate outcomes with different systems.

## AUTHOR CONTRIBUTIONS

**Laura de Girolamo** and **Elizaveta Kon**: Conceptualisation. **Laura de Girolamo** and **Elizaveta Kon**: Methodology. **Enrico Ragni**: Software. **Michela Taiana** and **Caterina Visconte**: Validation. **Enrico Ragni, Michela Taiana,** and **Caterina Visconte**: Formal analysis. **Enrico Ragni, Michela Taiana,** and **Caterina Visconte**: Investigation. **Laura Mangiavini** and **Giuseppe Peretti**: Resources. **Simona Landoni** and **Cecilia Colombo**: Data curation. **Enrico Ragni**: Writing—original draft preparation. **Laura de Girolamo**: Writing—review and editing. **Enrico Ragni**: Visualisation. **Laura de Girolamo** and **Elizaveta Kon**: Supervision. **Laura de Girolamo** and **Elizaveta Kon**: Project administration. **Laura de Girolamo** and **Elizaveta Kon**: Funding acquisition. All authors have read and agreed to the published version of the manuscript.

## CONFLICT OF INTEREST STATEMENT

The authors declare no conflicts of interest.

## ETHICS STATEMENT

The study was conducted in accordance with the Declaration of Helsinki, and approved by San Raffaele Hospital Ethics Committee (“Algoritmo decisionale per un approccio di medicina rigenerativa nel trattamento dell’ OA al ginocchio: nuovo processo basato sulla “profilazione” del paziente”, approval on date July 14, 2021 registered under number 253/2021). Informed consent was obtained from all subjects involved in the study.

## Supporting information

Figure S1. Flow cytometry gating strategy to identify ASCs in SVF and BMSCs in BMA and BMAC. A representative donor for each sample is shown.

Table S1. Demographic and haematological data for SVF, BMA and BMAC donors and samples.

Table S2. Soluble factors detected in BMAC and SVF samples ordered by differential amount within each category.

Table S3. Detected transcripts in inflamed chondrocytes treated with BMAC or SVF expressed as fold‐change vs untreated cells (CTRL) set as 1.

## Data Availability

The data sets generated and/or analysed during the current study are available in the OSF repository, https://osf.io/8dbex/?view_only=8eaa28c0da3449edac980572c64118a3.
