# Supplementary figures and images for: Side‐to‐side characterisation of cellular content, soluble factors and in vitro potential on chondrocytes for bone marrow aspirate concentrate and adipose‐derived stromal vascular fraction
Source: J Exp Orthop. 2025 May 12;12(2):e70254. doi: 10.1002/jeo2.70254 (PMC12066993; doi:10.1002/jeo2.70254)

A

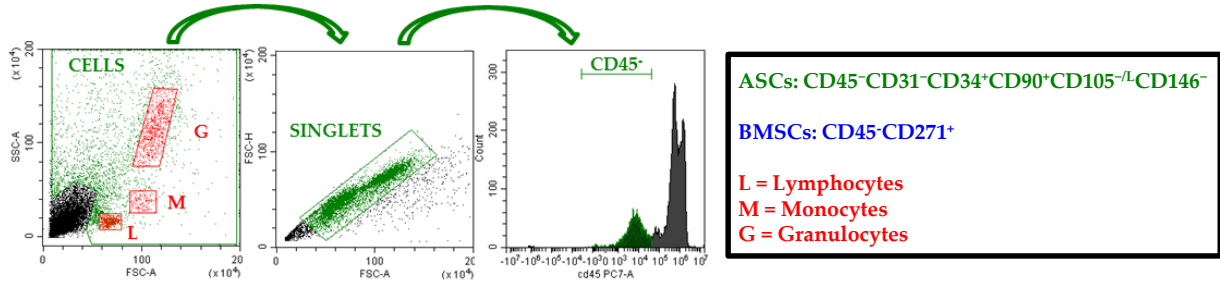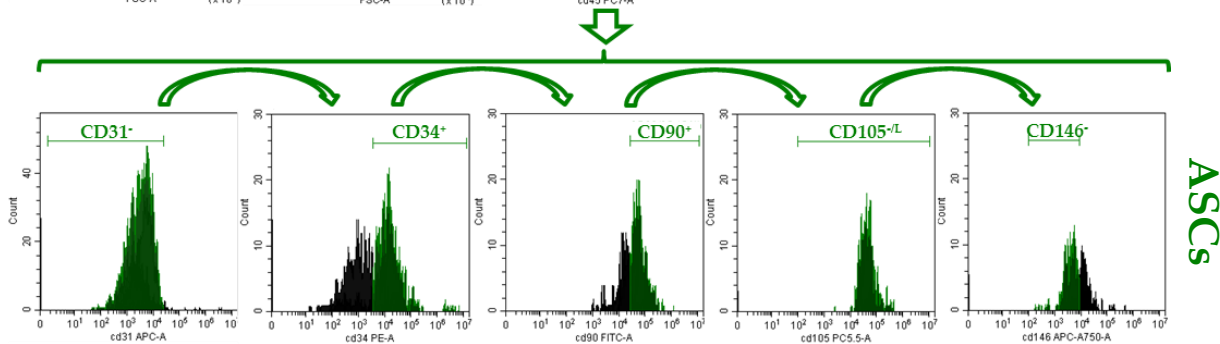

B

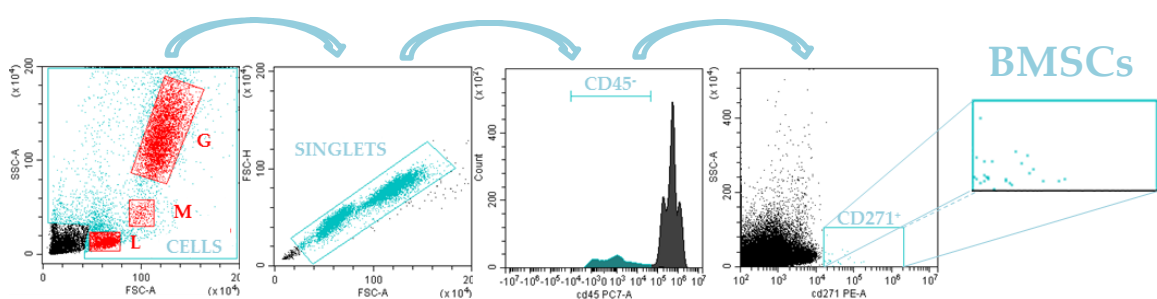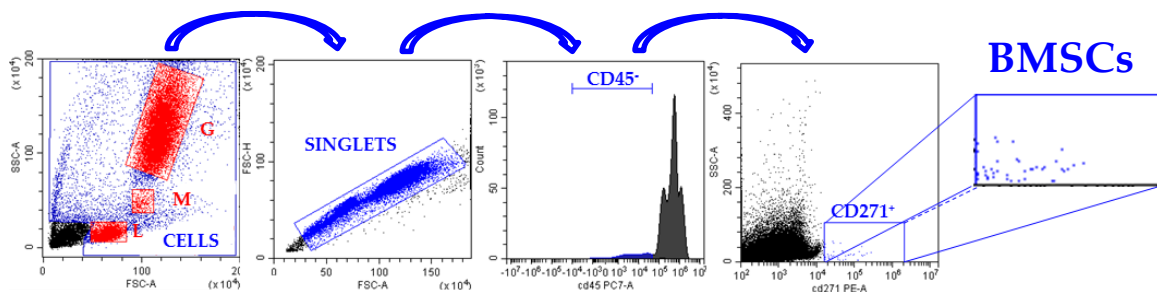

Supplement: Supplementary file 1 — Figure S1. Flow cytometry gating strategy to identify ASCs in SVF and BMSCs in BMA and BMAC. A representative donor for each sample is shown. [file JEO2-12-e70254-s004.pdf]
