# Supplementary material for: Side‐to‐side characterisation of cellular content, soluble factors and in vitro potential on chondrocytes for bone marrow aspirate concentrate and adipose‐derived stromal vascular fraction
Source: J Exp Orthop. 2025 May 12;12(2):e70254. doi: 10.1002/jeo2.70254 (PMC12066993; doi:10.1002/jeo2.70254)
Supplement: Supplementary file 2 — Table S1. Demographic and haematological data for SVF, BMA and BMAC donors and samples. [file JEO2-12-e70254-s003.pdf]

Supplementary Table S1. Demographic and haematological data for SVF, BMA and BMAC donors and samples.

A	SEX	AGE	BMI	KL	SVF WBC $10^3/\mu L$	SVF RBC $10^6/\mu L$	SVF PLT $10^3/\mu L$
S1	M	51	23	2	0.22	0.14	40
S2	F	42	28	2	0.08	0.03	6
S3	M	55	27	2	0.12	0.01	14
S4	M	57	28	3	0.17	0.01	10
S5	M	56	32	2	0.08	0.01	11
S6	F	69	36	2	0.04	0.01	8
S7	M	47	25	2	0.24	0.03	14
S8	F	50	25	2	0.33	0.09	14
S9	M	41	27	2	0.08	0.01	9
S10	F	56	22	2	0.47	0.00	0
S11	M	40	26	2	0.16	0.07	24
S12	M	51	27	2	0.07	0.09	18
S13	M	55	27	2	0.44	0.08	35
S14	M	61	27	2	0.16	0.03	16
S15	M	57	32	3	0.13	0.02	14
S16	M	54	25	2	0.80	0.26	12
S17	F	64	28	2	0.29	0.06	20
S18	M	52	26	2	0.24	0.15	25
S19	M	47	24	2	0.29	0.20	22
S20	F	44	29	2	0.46	0.11	22
S21	F	74	30	2	0.17	0.14	17
S22	M	68	28	3	0.51	0.07	25
S23	M	55	27	2	0.68	0.91	70
S24	M	61	24	3	0.19	0.01	9
S25	M	61	26	3	0.55	0.31	39
S26	M	48	25	3	0.64	0.10	20
S27	F	61	22	3	0.41	0.09	10
S28	F	43	28	2	0.14	0.01	10
S29	F	59	23	2	0.23	0.02	15
S30	F	61	26	2	0.33	0.08	23
S31	M	65	29	2	0.24	0.02	13
S32	F	72	27	3	0.5	0.03	13
S33	F	55	24	3	0.37	0.21	17
S34	M	71	28	2	0.48	0.09	21
S35	M	48	25	2	0.43	0.02	15
S36	M	51	27	3	0.09	0.03	25
S37	M	59	23	3	0.28	0.2	38
S38	M	73	26	3	0.31	0.42	42
S39	M	51	25	3	0.33	0.11	23

B	SEX	AGE	BMI	KL	BMA WBC $10^3/\mu L$	BMA RBC $10^6/\mu L$	BMA PLT $10^3/\mu L$	BMAC WBC $10^3/\mu L$	BMAC RBC $10^6/\mu L$	BMAC PLT $10^3/\mu L$
B1	M	53	28	3	7.83	3.51	129	40.21	3.74	351
B2	F	60	22	2	12.58	3.63	125	8.9	7.81	37
B3	M	39	30	2	15.73	3.03	48	52.74	3.39	55
B4	F	56	24	2	5.62	3.26	152	77.76	3.55	1072
B5	F	71	22	2	6.58	2.10	28	73.32	2.59	208
B6	M	49	26	3	12.67	3.72	108	50.87	6.34	311
B7	M	53	26	2	10.37	4.54	109	23.7	6.8	94
B8	M	47	26	2	9.88	4.57	72	-	-	-
B9	F	68	23	2	8.99	4.06	147	78.12	3.13	726
B10	M	39	34	2	17.65	4.00	250	123.23	3.92	1741
B11	F	55	22	2	3.68	2.64	44	61.61	5.89	936
B12	F	53	32	2	7.71	3.46	85	71.81	5.21	619
B13	M	58	25	2	6.85	4.30	43	22.84	6.69	39
B14	F	59	30	2	14.82	3.91	117	112.48	6.08	258
B15	F	63	33	2	6.62	3.99	153	62.32	4.22	1278
B16	M	46	24	2	13.89	3.85	55	75.15	5.27	84
B17	M	55	30	2	10.22	4.20	91	32.73	5.05	320
B18	F	57	32	3	17.25	3.78	137	115.93	5.41	446
B19	F	54	34	2	8.16	3.67	148	65.27	7.15	718
B20	F	65	26	2	10.52	3.93	165	77.24	4.48	780
B21	M	58	28	2	11.07	3.87	74	66.88	5.64	269
B22	M	50	28	3	5.58	4.13	133	43.8	6.89	685
B23	M	71	24	2	12.4	3.98	69	91.97	4.98	481
B24	M	67	30	2	16.87	4.25	128	63.27	6	559
B25	M	48	30	3	10.42	3.99	87	30.81	5.58	92
B26	M	44	24	3	7.57	4.14	172	34.86	6.37	554
B27	M	56	26	2	10.72	3.45	169	72	5.81	665
B28	M	55	30	3	-	-	-	100.88	6.04	291

"-"Coagulated sample. BMI for Body mass index. KL for Kellgren and Lawrence classification.
