# Supplementary material for: Side‐to‐side characterisation of cellular content, soluble factors and in vitro potential on chondrocytes for bone marrow aspirate concentrate and adipose‐derived stromal vascular fraction
Source: J Exp Orthop. 2025 May 12;12(2):e70254. doi: 10.1002/jeo2.70254 (PMC12066993; doi:10.1002/jeo2.70254)
Supplement: Supplementary file 3 — Table S2. Soluble factors detected in BMAC and SVF samples ordered by differential amount within each category. [file JEO2-12-e70254-s002.pdf]

Supplementary Table S2. Soluble factors detected in BMAC and SVF samples ordered by differential amount within each category.

CATEGORY	FACTOR	BMAC (N=8)		SVF (N=12)		BMAC/SVF	p-value
		pg/ml	SD	pg/ml	SD		
	CCL16	1206	628	28	16	43.7	<0.0001
	CCL23	190	137	18	17	10.6	<0.0001
	SPP1	5344	5648	523	725	10.2	0.0002
	CXCL16	1739	1077	387	191	4.5	<0.0001
	CCL18	1309	516	436	130	3.0	<0.0001
	PPBP	476	220	172	59	2.8	0.0002
	CCL14	591	230	223	56	2.7	<0.0001
	PF4	28658	11070	11477	2262	2.5	<0.0001
CHEMOKINES	IL9	67300	21535	32082	26311	2.1	0.0011
	CXCL11	93	88	47	61	2.0	
	AXL	739	481	445	168	1.7	
	CCL27	384	284	221	315	1.7	
	CXCL10	88	27	55	91	1.6	
	CCL17	45	14	30	49	1.5	
	CCL13	44	13	39	69	1.1	
	LIF	23	7	21	40	1.1	
	MIF	3919	5288	3452	641	1.1	
	PDGFB	3806	2036	116	87	32.7	<0.0001
	AGRP	347	157	25	26	13.7	<0.0001
	SERPINE1	5979	4207	440	303	13.6	<0.0001
	TEK	7043	3521	717	305	9.8	<0.0001
	ANG	578	229	60	108	9.6	0.0002
	IL2RB	5624	4366	595	266	9.5	0.0008
	IL2RA	1104	613	117	56	9.4	<0.0001
	PLG	109300	31967	12267	10306	8.9	<0.0001
	IL23A	35155	15550	4133	2294	8.5	<0.0001
	TREM1	3917	1722	505	325	7.7	<0.0001
	RETN	4997	2774	662	439	7.5	<0.0001
	CXCL12	1114	435	170	98	6.6	<0.0001
	IL6ST	33462	26579	5302	2814	6.3	<0.0001
CYTOKINES	TDGF1	396	166	66	41	6.0	<0.0001
	SIGLEC5	6275	1682	1285	667	4.9	<0.0001
	CDH1	48708	15739	11141	5208	4.4	<0.0001
	TGFB2	627	275	151	64	4.2	<0.0001
	ICAM2	23226	5499	5838	1965	4.0	<0.0001
	NRCAM	695	408	176	122	3.9	0.0002
	SHH	44	21	12	7	3.7	0.0001
	IL13RA1	2692	1014	754	191	3.6	<0.0001
	TNFRSF10D	418	400	117	97	3.6	0.0159
	IL17B	2707	1129	779	449	3.5	<0.0001
	FASLG	222	338	65	44	3.4	0.0557
	TGFB1	2259	1202	672	310	3.4	0.0003
	FLT1	10339	7707	3286	2201	3.1	0.0022
	INHBA	915	260	303	23	3.0	<0.0001
	DKK1	840	458	331	121	2.5	<0.0001

	CD40	419	145	169	73	2.5	<0.0001
	ANGPT1	9603	3090	4313	3464	2.2	0.0015
	CTSS	1515	662	725	306	2.1	0.0019
	VEGFC	192	72	110	71	1.7	
	LGALS7	890	833	556	544	1.6	
	THPO	12829	9920	8488	4700	1.5	
	IL13RA2	1751	1891	1491	1511	1.2	
	FCGR2B	695	269	1154	836	0.6	
	FST	345	137	615	250	0.6	
GROWTH FACTORS	BDNF	59243	29470	1510	768	39.2	<0.0001
	IGFBP3	13954	8289	985	496	14.2	<0.0001
	KITLG	46813	55072	4136	2715	11.3	0.0015
	PDGFA	724927	246085	64795	38247	11.2	<0.0001
	KIT	1255396	524734	177664	123198	7.1	<0.0001
	EGFR	298621	80945	53415	20423	5.6	<0.0001
	EGF	748	529	141	65	5.3	<0.0001
	TNFRSF11B	10715	9887	2392	1601	4.5	0.0007
	TGFB3	686	215	155	85	4.4	<0.0001
	IGFBP2	39947	14924	9377	5995	4.3	<0.0001
	NGF	18471	7072	4413	2306	4.2	<0.0001
	NGFR	25041	32443	6226	7968	4.0	0.0252
	TGFA	61885	82513	15390	11381	4.0	0.0055
	GDF15	25227	13538	6327	2360	4.0	0.0001
	GH1	21220	8770	5502	2931	3.9	<0.0001
	HBEGF	8281	9176	2151	1802	3.8	0.0159
	AREG	20088	9896	5450	2605	3.7	0.0001
	IGF1	50990	29804	13844	6240	3.7	0.0005
	NTF4	25658	15971	7119	2247	3.6	0.0008
	FLT4	29279	8926	8696	3648	3.4	<0.0001
	CSF1R	1097528	328374	335263	119404	3.3	<0.0001
	FIGF	42228	32774	13170	6327	3.2	0.0015
	NTF3	20903	10775	6583	2853	3.2	0.0003
	BMP7	58585	26214	18930	8299	3.1	0.0002
	FGF4	98237	35021	33001	13057	3.0	<0.0001
	FGF7	10881	3183	3741	1543	2.9	<0.0001
	IGFBP6	17706	8073	6126	2602	2.9	0.0002
	PROK1	72377	32388	25517	8650	2.8	0.0001
	GDNF	4254	1102	1530	1075	2.8	0.0005
	PGF	5169	2026	1863	843	2.8	<0.0001
	BMP4	5717	3575	2090	1722	2.7	0.0007
	BMP5	173751	64691	73288	34813	2.4	0.0002
	VEGFA	19184	7154	8453	4099	2.3	0.0005
	IGFBP4	14152	6370	7761	5206	1.8	
	FGF2	45687	9277	38247	16667	1.2	
	IGFBP1	2456	795	1973	1754	1.2	
	HGF	15322	10866	195626	232456	0.1	<0.0001
INFLAMMATION	TIMP1	17601	3083	21114	18124	0.8	
	CCL5	11243	3371	14768	14730	0.8	
	ICAM1	7012	2756	8970	7202	0.8	

	IL6R	6171	1092	8696	7999	0.7	
	CCL11	621	734	1225	1729	0.5	
	TIMP2	25592	8141	67300	56497	0.4	
	TNFRSF1B	3959	3174	11865	12040	0.3	
RECEPTORS	ERBB3	410	365	30	14	13.5	0.0018
	VCAM1	71624	32889	6965	5669	10.3	<0.0001
	SELL	55810	18465	7638	5518	7.3	<0.0001
	TNFRSF10C	2982	2164	430	483	6.9	<0.0001
	MOK	688	420	122	71	5.6	0.0002
	ALCAM	2403	1125	544	253	4.4	<0.0001
	TNFRSF17	1783	899	495	154	3.6	0.0001
	CEACAM1	189	130	56	26	3.4	0.0026
	SELE	1698	621	516	232	3.3	<0.0001
	PLAUR	980	449	324	120	3.0	0.0001
	FAS	124	55	42	22	3.0	0.0002
	PI3	1092	322	399	184	2.7	<0.0001
	ENG	132	76	52	22	2.5	0.0029
	LCN2	2138	986	869	218	2.5	0.0004
	CD14	9444	3563	3872	849	2.4	<0.0001
	TNFRSF9	136	106	70	41	1.9	
	CD40LG	401	230	228	79	1.8	
	TYRO3	522	264	317	192	1.6	
	LYVE1	2201	1003	1376	359	1.6	
	TNFRSF14	280	140	193	78	1.5	
	TNFRSF21	1838	923	1504	460	1.2	
	PECAM1	3120	1675	3322	1313	0.9	
	FLT3LG	22	12	38	12	0.6	

A fold change at least ≥ 2 or ≤ 0.5 was used to calculate the p-value that is shown only when ≤ 0.05.
