# Supplementary material for: Side‐to‐side characterisation of cellular content, soluble factors and in vitro potential on chondrocytes for bone marrow aspirate concentrate and adipose‐derived stromal vascular fraction
Source: J Exp Orthop. 2025 May 12;12(2):e70254. doi: 10.1002/jeo2.70254 (PMC12066993; doi:10.1002/jeo2.70254)
Supplement: Supplementary file 4 — Table S3. Detected transcripts in inflamed chondrocytes treated with BMAC or SVF expressed as fold‐change vs untreated cells (CTRL) set as 1. [file JEO2-12-e70254-s001.pdf]

Supplementary Table S3. Detected transcripts in inflamed chondrocytes treated with BMAC or SVF expressed as fold-change *vs* untreated cells (CTRL) set as 1.

	**IL1β *vs* untreated**			**IL1β+BMAC *vs* untreated**			**IL1β+SVF *vs* untreated**		
	FOLD	SD	**P-VALUE**	**FOLD**	**SD**	**P-VALUE**	**FOLD**	**SD**	**P-VALUE**
CXCL8	88.11	18.06	****	26.46	8.76		76.93	27.97	****
CXCL2	56.36	10.22	****	7.94	7.49		25.70	11.37	**
IL6	49.98	15.10	****	3.58	2.73		12.88	8.23	*
CCL20	37.02	15.93	****	9.85	4.47		31.64	13.37	****
CXCL1	36.97	8.62	****	5.65	5.64		20.07	9.89	**
CSF2	23.03	11.40	****	7.05	3.84		11.06	7.91	*
THPO	8.20	13.56		8.38	12.52		3.68	2.51	
CXCL5	4.62	4.00	**	4.19	8.41		1.84	1.41	
IL1B	4.47	1.52	****	0.80	0.38		2.49	0.87	*
IL22	3.29	2.08		1.31	0.94		4.70	10.42	
CNTF	2.45	1.27	*	1.35	0.92		1.68	0.74	
CX3CL1	2.32	2.25		0.76	0.72		1.08	0.96	
CCL7	2.20	3.00		1.90	2.66		1.79	1.17	
CXCL16	2.13	0.50	*	1.69	0.67		2.02	0.72	*
CSF3	1.72	0.90		0.84	0.53		1.21	1.47	
IL27	1.65	1.09		1.72	1.48		1.33	0.92	
SPP1	1.60	0.39		1.49	0.67		1.59	0.94	
IL15	1.55	0.92		1.26	1.01		1.70	0.67	
CSF1	1.24	0.22		0.57	0.29		0.82	0.23	
MIF	1.17	0.23		1.39	0.76		1.04	0.21	
LIF	1.13	0.54		0.44	0.21	*	1.11	0.38	
GPI	1.11	0.23		1.50	0.30		1.16	0.11	
IL18	1.08	1.08		0.49	0.40	*	0.46	0.45	*
C5	1.05	0.30		0.37	0.39		0.63	0.39	
IL1A	1.03	0.54		0.59	0.25		0.82	0.27	
IL12A	0.89	0.21		0.91	0.14		0.96	0.26	
BMP6	0.69	0.13		1.05	0.55		1.04	0.28	
TNFSF10	0.63	0.51		0.17	0.11	***	0.23	0.12	**
IL13	0.60	0.49		1.66	1.88		0.95	0.58	
TGFB2	0.58	0.09		0.42	0.11	****	0.46	0.09	****
TNFSF11	0.41	0.70	*	2.30	3.66		0.36	0.39	
CCL1	0.39	0.09		1.77	1.18		1.17	1.29	

A fold change *vs* CTRL at least ≥ 2 or ≤ 0.5 was used to calculate the p-value that is shown only when ≤ 0.05. * for p-value ≤ 0.05, ** ≤ 0.01, *** ≤ 0.001, **** ≤ 0.0001.
